# Capacity of State and Territorial Health Agencies to Prevent Foodborne Illness

**DOI:** 10.3201/eid1101.040334

**Published:** 2005-01

**Authors:** Richard E. Hoffman, Jesse Greenblatt, Bela T. Matyas, Donald J. Sharp, Emilio Esteban, Knachelle Hodge, Arthur Liang

**Affiliations:** * Council of State and Territorial Epidemiologists, Atlanta, Georgia, USA; † New Hampshire Department of Health and Human Services, Concord, New Hampshire, USA; ‡ Massachusetts Department of Public Health, Boston, Massachusetts, USA; § Centers for Disease Control and Prevention, Atlanta, Georgia, USA; ¶ U.S. Department of Agriculture, Alameda, California, USA

**Keywords:** food poisoning, epidemiology, public health surveillance, disease outbreaks, research

## Abstract

The capacity of state and territorial health departments to investigate foodborne diseases was assessed by the Council of State and Territorial Epidemiologists from 2001 to 2002 with a self-administered, web-based survey. Forty-eight health departments responded (47 states and 1 territory). The primary reason for not conducting more active case surveillance of enteric disease is lack of staff, while the primary reasons for not investigating foodborne disease outbreaks are limited staff and delayed notification of the outbreak. Sixty-four percent of respondents have the capacity to conduct analytic epidemiologic investigations. States receiving Emerging Infections Program (EIP) funding from the Centers for Disease Control and Prevention more often reported having a dedicated foodborne disease epidemiologist and the capability to perform analytic studies than non-EIP states. We conclude that by addressing shortages in the number of dedicated personnel and reducing delays in reporting, the capacity of state health departments to respond to foodborne disease can be improved.

## Abstract 2

Foodborne disease reporting can increase through infrastructure improvements.

Foodborne illnesses are common. Each year an estimated 76 million foodborne illnesses occur, with 325,000 hospitalizations and 5,000 deaths (*1*), and a recent estimate of annual costs for medical treatment, productivity loss, and premature deaths resulting from these illnesses is $6.5 billion (*2*). The National Food Safety Initiative (NFSI) was started in 1997 as an effort to decrease the incidence and risk for foodborne illness (*3*). The NFSI ended in 2001, but at the Centers for Disease Control and Prevention (CDC), the former NFSI funding and activities have been institutionalized as an ongoing food safety program. Continued progress on the part of regulators and industry to improve food safety are dependent on local, state, and federal agencies’ ability to conduct epidemiologic and laboratory investigations that identify the offending agents and link them with specific foods.

Improvements in detecting and investigating foodborne illnesses were made during the 1990s when CDC implemented the Foodborne Diseases Active Surveillance Network (FoodNet), a component of the Emerging Infections Programs (EIP), and PulseNet (*4*,*5*). EIP is a network of epidemiology programs in state health departments that is funded and coordinated by CDC. It is intended to be a national resource for surveillance and epidemiologic research that goes beyond the routine public health department functions. Active, laboratory-based surveillance is the foundation of 2 core EIP projects conducted at all sites: Active Bacterial Core Surveillance and Foodborne Disease Active Surveillance. Ten states currently receive EIP support from CDC. PulseNet, unlike EIP, is intended to be a national molecular subtyping network for foodborne disease surveillance. It was established by the CDC in 1996 to facilitate subtyping bacterial foodborne pathogens by state health department laboratories. Even after implementing FoodNet and PulseNet, much work remains to improve the state and local public health agencies’ capacity to detect and investigate foodborne disease.

In 1999, CDC provided funding to both Council of State and Territorial Epidemiologists (CSTE) and the Association of Public Health Laboratories (APHL) to conduct assessments of states’ foodborne disease investigation capacity. The purpose of both assessments was to determine priorities for improving food safety program support. The CSTE assessment was intended to concentrate primarily on state and territory health departments’ capacity to monitor and investigate foodborne illness. This report presents the results of the CSTE survey, which was conducted from October 2001 to March 2002, of 48 state and territorial health agencies,

An expert CSTE committee, composed of state and local epidemiologists from Colorado; Philadelphia; and Los Angeles County, California; an environmentalist from DeKalb County, Georgia; a state laboratorian from Rhode Island; staff from the CDC’s National Center for Infectious Disease, Division of Bacterial and Mycotic Diseases-Food Safety Office; and CSTE staff from its national office developed a survey instrument that was pilot-tested in 6 states and subsequently revised. The final instrument consisted of 106 questions. We present analyses of selected questions, a complete tabulation of all results, and all qualitative responses. Questionnaires are available from the CSTE website (*6*). The data can be used as a baseline reference for future surveys of state and territorial capacity to investigate foodborne disease.

## Methods

The assessment instrument was a self-administered, Web-based survey. Respondents were state and territorial epidemiologists with knowledge in the area of foodborne diseases. The assessment was conducted from October 2001 through February 2002, and during the 4-month survey period, reminder telephone calls and emails were made from the CSTE national office to health agencies that had not yet responded.

The instrument’s 106 questions covered background information about the responding agency, epidemiologic surveillance capacity to identify sporadic and outbreak-related illnesses; capacity to investigate and respond to outbreaks; public health infrastructure to support food safety activities, defined as staffing, facilities, equipment, supplies, information, communication between epidemiology and laboratory units, and education and training of staff; and legal authority of the agency. We restricted results in this article to questions pertaining to agency capacity and operations, barriers to the investigation of foodborne diseases, and staffing of the epidemiology program (a subset of “barriers”).

Forty-eight health departments responded (47 states [response rate = 94%] and 1 territory [Guam]); Pennsylvania, Illinois, Nevada, and Puerto Rico did not submit responses. Some questions did not elicit 48 responses. Responses reflect the perspective of the epidemiology program in the agency. The frequency and percentage for each response were calculated on the basis of the total number of responses to that question. Percentages are rounded to the nearest integer. The phrasing of questions in tables in the Results section has, in some instances, been shortened from the exact words used in the questionnaire.

We also examined responses by whether the responding agency received EIP funding from CDC (8 of 9 EIP sites that were funded at the time responded to the survey: Colorado, Connecticut, Georgia, New York, Minnesota, Oregon, Tennessee, and California/San Francisco Bay) and whether the responding agency was a jurisdiction with large population (10 largest population states in 2000 U.S. census; number of respondents = 8; population range 33,871,648–8,186,453), medium population (states ranked 11th to 20th in population in 2000 census; number of respondents = 10; population range 8,049,313–5,130,632), or small population (the remaining states and 1 territory; number of respondents = 30; population range 4,919,479–154,805). The term “small population states” includes 29 states and 1 territory (Guam). The 8 responding EIP sites included 3 large, 1 medium, and 4 smaller population states.

## Results

Forty percent of the states receive laboratory reports electronically. The primary reason reported for not conducting more active case surveillance is lack of staff. The primary reasons reported for not investigating foodborne disease outbreaks are limited staff and delayed reporting of the outbreak. Sixty-four percent of respondents have the capacity to conduct analytic epidemiologic investigations. Thirty-five percent of respondents have a protocol to guarantee chain of custody for food specimens. Eighty-one percent of respondents can obtain public health laboratory, environmental health, and sanitation support 24 hours per day. Fifty-four percent of respondents have broadcast fax or email capability to hospital emergency rooms and to physicians (Tables 1,2,3).

**Table 1:** Capacity and operations of epidemiologic, laboratory, and environmental programs in state and territorial health departments

Question	n	% yes	% no	% not sure
Does the Epidemiology Office have the ability to receive electronic laboratory reporting of enteric diseases?	48	40	60	
Do you routinely collect stool samples for testing?	47	98	2	
Do you routinely collect vomitus for testing?	47	38	62	
Do you have broadcast fax or email capability to: (list all that apply)	48			
Other health departments within the state		88		
Hospital infection control specialists		77		
Hospital emergency rooms		54		
Physicians		50		
Other state health departments		40		
Other		19		
Do you conduct syndromic surveillance for diarrheal disease?	48	15	77	8
Do you have adequate capacity to conduct an analytic epidemiologic investigation, i.e., case-control or cohort studies?	47	64	30	6
Does your agency have legal authority to:				
Collect reports of suspected enteric diseases?	48	90	4	6
Perform on-the-spot emergency environmental/sanitation inspections?	48	85	4	10
Exclude sick or infected employees from food handling duties?	48	83	13	4
Share information related to foodborne outbreaks with federal agencies, e.g., USDA, FDA, and CDC?*	47	83	4	13
Close a food service facility?	48	81	15	4
Collect reports of clinical syndromes?	48	71	19	10
Embargo or condemn food?	47	66	11	23
Is there a regulation/statute specifically requiring submission of certain enteric isolates to the public health laboratory?	48	54	38	8
Does the department of health have a protocol to guarantee chain of custody for food environmental specimens?	48	35	48	17

*USDA, United States Department of Agriculture; FDA, Food and Drug Administration; CDC, Centers for Disease Control and Prevention.

**Table 2:** Barriers to investigating foodborne disease in state and territorial health departments

Question	n	% yes	% no	% not sure
Of the outbreaks that are not investigated, which factors most limit your ability to investigate? (list all that apply)	48			
Delayed notification		83		
Limited staff		67		
Lack of apparent importance		46		
Laboratory capacity		21		
Jurisdictional issues		19		
Political consideration		13		
Expertise		13		
Other		13		
Travel policy constraints		11		
Statistical support		8		
Ability to pay overtime		8		
In outbreaks in which food specimens were not submitted, what were the barriers to laboratory testing?	47			
Leftovers not available		98		
Wrong food collected		32		
Unnecessary		17		
Other		13		
No capability for food testing, i.e., laboratory equipment		11		
Insufficient expertise at laboratory		6		
Too expensive		4		
Do you feel there are barriers for conducting more active case surveillance?	48	88	8	4
If yes, which of the following reasons apply: (list all that apply)	42			
Lack of staff		81		
Too time-consuming		60		
Other		33		
Low priority		29		
Lack of expertise		12		

**Table 3:** Staffing in epidemiology programs for foodborne disease surveillance and investigation

Question	n	% yes	% no	% not sure
For sporadic cases, do you have enough people to:	48			
Compare to standardized case definition		85	15	
Enter data		79	19	2
Review data for consistency and completeness		71	27	2
During outbreaks, do you have enough people to:	48			
Compare to standardized case definition		90	10	
Enter data		73	19	8
Review data for completeness and consistency		71	23	6
In your enteric/foodborne disease epidemiology program, do you have sufficient statistical support?	47	45	47	9
Do you have a dedicated enteric/foodborne disease epidemiologist at your agency?	48	48	50	2
If yes to question above, what is the highest level of education of the epidemiologist?	23			
Masters degree		61		
Doctoral degree		26		
Bachelor degree		13		
During an outbreak investigation, do epidemiologists routinely accompany environmental health/sanitation specialist(s)?	48	44	50	6
Is there a 24-hour on-call response mechanism for foodborne disease issues?	48	96	4	
Can you get public health laboratory support 24/7/365?	48	81	15	4
Can you get environmental health/sanitation support 24 hours per day?	48	60	23	17
Do your epidemiologists receive training in environmental food facility inspections?	48	13	85	2
Do your environmental health/sanitation specialists receive training in epidemiology?	48	63	33	4

We did not find that EIP sites always reported more capacity and more advanced operations than non-EIP sites. A greater percentage of EIP sites than non-EIP sites reported adequate capacity to conduct analytic epidemiologic studies (88% vs. 59%) and having a regulation or statute specifically requiring the submission of certain enteric isolates to the public health laboratory (75% vs. 50%). On the other hand, a smaller percentage of EIP sites than non-EIP sites reported having the capacity to broadcast faxes to hospital emergency departments (50% vs. 55%) and to conduct syndromic surveillance for diarrheal disease (0% vs. 18%). The percentage of EIP sites having a protocol to guarantee chain of custody for food environmental specimens was nearly the same as for non-EIP sites (38% vs. 36%).

Likewise, we found that large population states did not consistently have more capacity and more advanced operations than medium or small population states or territories. Seventy-five percent of large states, 90% of medium states, and 52% of small population states reported adequate capacity to perform analytic epidemiologic studies. Thirty-eight percent of large states, 30% of medium, and 67% of small states reported the capacity to broadcast fax to hospital emergency departments. The differences between state size and having a chain of custody protocol for food specimens were relatively small (50% of large, 40% of medium, and 30% of small population states), while the differences in percentage reporting a legal requirement to submit certain enteric isolates to the public health laboratory were relatively large: 38% of large states, 70% of medium states, and 53% of small states.

As for factors that limit ability to investigate outbreaks, the most common reason given by both EIP and non-EIP sites was “delayed notification” (88% vs 83%). The percentage of EIP sites and non-EIP sites reporting “limited staff” (63% vs 68%) and “lack of importance” (50% vs 45%) were similar. Delayed notification was the most frequent reason given by large (75%), medium (100%), and small (80%) population states for not investigating outbreaks. Seventy percent of small states compared to 70% of medium states and 50% of large states reported limited staff as a reason for not investigating outbreaks.

Seventy-two percent of EIP sites versus 83% of non-EIP sites reported having laboratory support 24 hours per day, whereas 75% of EIP sites compared to 43% of non-EIP sites reported having a dedicated enteric/foodborne epidemiologist. For these same two questions, 73% of small population states versus 100% of large and 90% of medium states had laboratory support 24 hours per day, and 75% of large and 80% of medium states had a dedicated enteric/foodborne disease epidemiologist compared to 30% of small population states. Lastly, during outbreaks, 100% of EIP sites versus 68% of non-EIP sites reported that they had enough people to enter data. For this question, the differences between large, medium, and small population states were relatively small (88%, 70%, 70%, respectively).

## Discussion and Conclusion

In the United States, the primary responsibility for foodborne disease surveillance and investigation lies with state, territorial, and local health agencies, with technical backup and funding support from CDC and other federal agencies, including the Food and Drug Administration and the Food Safety and Inspection Service of the U.S. Department of Agriculture. Within a state public health agency, reducing the incidence of foodborne disease requires a sensitive surveillance system, timely epidemiologic investigation of sporadic cases and outbreaks with the most current laboratory technologies, and coordination of epidemiology, environmental, and laboratory programs.

This report is the fourth in a series by CSTE to assess epidemiologic capacity in state and territorial health departments. The 3 previously published surveys concerned overall capacity and maternal and child health capacity (*7*–*10*). While infectious disease capacity was addressed in the overall survey conducted from November 2001 through April 2002, this report is the most detailed analysis of states’ and territories’ foodborne disease capacity to date. The findings in the overall capacity report concerning reasons why outbreaks were not investigated by the state health department are similar to findings in our report: of 42 respondents 40 (95%) reported delayed notification of case reports, 33 (79%) reported limited staff, and 31 (74%) reported competing priorities for use of public health resources (*7*).

In the areas of foodborne disease surveillance and investigation, our report documents that the aggregate perception of a large sample of epidemiologic leaders in state and territorial health departments is that, as of 2002, more resources were needed. The data are self-reported and do not include responses from a few large states and Puerto Rico. The survey found that lack of staff was the most frequent reason (81% of respondents) for not conducting more active case surveillance, and the most frequent reasons given for not investigating outbreaks were delayed notification (83%) and limited staff (63%) (Table 2).

Our findings are also consistent with a 50-state survey conducted by the General Accounting Office in 2000 to 2001 (*2*). That survey found, for example, 32 (64%) of 50 states indicated that more trained epidemiologists were needed at the state level to investigate outbreaks, and 44 (88%) of 50 states indicated that more trained epidemiologists were needed at the local level to investigate outbreaks.

If a state or territory had more epidemiologists to conduct surveillance, fewer delays would likely occur in recognition of outbreaks, and more expertise would be available to conduct investigations. Thus, by addressing shortages in the number of dedicated personnel and reducing delays in reporting, the capacity of state health departments to respond to foodborne disease can be improved.

We also performed comparisons of EIP to non-EIP sites and of large, medium, and small population states. Only 8 of 10 possible large population states and 8 of 9 EIP states were included, so the analyses must be interpreted cautiously. Because these comparisons were conceived after the survey data had been collected, we did not perform analytic statistical tests, which could be misinterpreted. Our descriptive findings are presented for interest and generation of hypotheses. We observed that EIP sites more frequently stated they had a dedicated foodborne disease epidemiologist, the capacity to perform analytic epidemiologic studies, and sufficient personnel to enter data during an outbreak than non-EIP sites. These findings would be expected, however, because 1 of the 2 core EIP projects is FoodNet. In other measures of capacity and program structure not specifically funded by the EIP programs, such as on-call laboratory support, not much difference existed between EIP and non-EIP sites.

The findings of this report do not indicate the quantity of resources needed to ensure sufficient capacity to protect the nation, and do the survey results do not direct the allocation of new resources. One approach to this issue would be to assess the reported incidence of enteric disease and foodborne outbreaks with respect to self-reported capacity to monitor and investigate foodborne disease. However, the nation’s system for identifying, investigating, and reporting foodborne diseases has not produced consistent and reliable data of adequate quality to perform such analyses. For example, in 1997, a total of 27 states and 3 territories reported zero outbreaks (10). More outbreaks must have occurred than were reported. Whatever the various reasons for such underreporting, the existing surveillance data are insufficient for addressing programmatic issues, such as where to invest in the public health system and what improvements in public health may reasonably be expected from such investment. Nevertheless, analyses are not needed to justify that every state and territory needs 24 hours per day epidemiologic, laboratory, and environmental health and sanitation on-call response capacity, as well as the capacity to communicate with public health and medical care providers, policymakers, and the public.

The analyses in this report provide a picture of the status of the nation at a time just before the distribution in 2002 of more than $1 billion to state, territorial, and local health agencies to improve bioterrorism response and preparedness capacity. Several criteria exist for the mitigation of foodborne illness listed in the bioterrorism preparedness cooperative agreement award notice and grant guidelines (Procurement and Grants Office, CDC, Announcement No. 99051). For example, having a formal outbreak investigation team is an illustration of focus area A (preparedness planning and readiness) of the bioterrorism preparedness cooperative agreement criteria; 70% of the respondents reported having this capacity. One of the guidelines in focus area G (education and training) is financial support by the state health agency for enteric disease and foodborne illness continuing education; more than half of the respondents in this survey reported that their agency provides this financial support. Although only 54% of states and territories reported that they could send broadcast faxes of health information to emergency departments, this particular capacity is a high priority for bioterrorism preparedness and is almost certain to have been further improved since the survey was completed.

In addition to the food safety minimum performance and capacity standards for epidemiology and surveillance adopted by CSTE as a position statement in 2003 (*11*), we recommend that for the short-term, objective measures of foodborne disease surveillance, reporting, and investigation be developed by local, state, and federal agencies. For example, the intervals from enteric disease onset until the case is reported to CDC may be measured in each state agency. Such measures can be used to indicate areas of need, to document areas of improvement, and to support the appropriation of new funds and the allocation of resources in lieu of enteric disease incidence.

This survey and the surveys of overall and maternal and child health epidemiologic capacity demonstrate a need for a larger workforce of epidemiologists. In response to the surveys, CSTE convened a workforce summit of leaders from within the CSTE organization, CDC, the Association of State and Territorial Health Officers, the American Public Health Association, and the Association of Schools of Public Health in January 2004 (*12*). In addition, at its annual meeting held in June 2004, the CSTE membership approved a resolution calling for an annual National Epidemiologist Awareness Day to bring attention to the work of epidemiologists in protecting the nation’s health (*12*). While this report and the mentioned activities of CSTE are specific to disease prevention by states and territories in the United States, similar capacities may be needed by public health agencies in other regions of the world, such as the European Union and the W.H.O. Global Salm-Surv programme. We hope that the survey design and the results will provide guidance and comparisons for readers in other countries.
